# Sulphuric Acid *vs.* Epistaxis and Stinking Feet

**Published:** 1877-05

**Authors:** Jno. J. Taylor

**Affiliations:** Streator, Ill.


### SULPHURIC ACID *vs.* EPISTAXIS AND STINKING FEET.

By JNO. J. TAYLOR, M. D., Streator, Ill,

It gives me pleasure to be able to call the attention of the profession to the use of sulphuric acid in the treatment of "nose bleed" and stinking feet.

The acid acts kindly and promptly in all cases not connected with a malignant taint, or where due to polypus, and especially in individuals at or near the age of puberty.

Even in polypus, after its removal, the acid is beneficial.

Its action is accounted for in several ways, first, perhaps, because the blood is rapidly thickened,—made more plastic or subalkaline. It also acts as an astringent and tonic, giving tone to the weakened vessels and to the general system.

Its use requires some care in cases where hemorrhage is very free and frequent, as a sudden arrest sometimes produces unpleasant head symptoms. My plan is to prescribe the acid *two to four times a day in sufficient quantity to make half a glass of sweetened water, quite tart* to the *patient's taste.*

At first, the patient will crave the drink much more tart than after a few days. After giving it three or four days, whether unpleasant head symptoms come on or not, it is best to discontinue its use a couple of days. It seldom becomes necessary to repeat its use after a couple of weeks.

There is one other use to which sulphuric acid is of special benefit. I mean stinking, sweating feet.

Given two or three times a day, as in nose bleeding, the patient will quickly be relieved of this unpleasant affection. In this class of cases, however, I frequently find it necessary to give some mild ferruginous preparation, such as the Ferri et Potassæ Tartras, in sherry wine—5 to 7 grs. a dose. The latter should be given just after dinner, and the acid in the morning and evening, and on an empty stomach.

